# Social network size and mental health among older adults in Japan: A nationwide cross-sectional analysis of the JACSIS 2024 study

**DOI:** 10.1016/j.ssmph.2026.101943

**Published:** 2026-06-24

**Authors:** Kazumi Kubota, Miya Aishima, Yukiko Matsumura, Takeshi Miura, Maya Minamizaki, Yu Koizumi, Yuka Kanoya, Takahiro Tabuchi

**Affiliations:** a Shimonoseki City University, Shimonoseki, Yamaguchi, Japan; b Department of Women's Health Population Sciences, National Center for Child Health and Development, Setagaya-ku, Tokyo, Japan; c Department of Healthcare Information Management, The University of Tokyo Hospital, Bunkyo-ku, Tokyo, Japan; d Department of Nursing, Shimonoseki City University, Shimonoseki, Yamaguchi, Japan; e Department of Gerontological Nursing, School of Medicine, Yokohama City University, Yokohama, Kanagawa, Japan; f Division of Epidemiology, School of Public Health, Graduate School of Medicine, Tohoku University, Sendai, Miyagi, Japan

**Keywords:** Healthy ageing, Social networks, Older adults, Psychological distress, Japan, Lubben social network scale

## Abstract

**Introduction:**

Social relationships are crucial for healthy ageing, but the role of social network size in older adults’ mental health remains underexplored in nationwide studies. We examined associations between social network size and mental health in a national sample of older Japanese adults.

**Methods:**

We analysed cross-sectional data from the 2024 Japan COVID-19 and Society Internet Survey (JACSIS), a nationwide internet survey, including 6786 respondents aged 65–84 years. Mental health outcomes were poor mental health days in the past 30 days (any vs none; ≥7 vs < 7 days) and psychological distress (moderate: K6 ≥5; severe: K6 ≥13). Social network size was measured using the Lubben Social Network Scale (LSNS-6: family/friends; LSNS-8: including professional contacts) and categorized into tertiles. Logistic regression estimated odds ratios (ORs) adjusted for demographic, socioeconomic, health, lifestyle, and work-related factors.

**Results:**

Overall, 11.8% reported any poor mental health day, 16.3% had moderate distress, and 1.9% had severe distress. In fully adjusted models, smaller social network size was consistently associated with poorer mental health in graded patterns. Compared with the highest LSNS-6 tertile, the lowest tertile had lower odds of no poor mental health days (OR = 0.80, 95% CI: 0.70–0.92) and higher odds of moderate (OR = 2.74, 95% CI: 2.28–3.29) and severe distress (OR = 6.54, 95% CI: 3.42–12.51). Similar patterns were observed for LSNS-8.

**Conclusions:**

Larger social networks were associated with fewer poor mental health days and lower psychological distress among older Japanese adults.

## Highlights

• Examined social network size and mental health among older Japanese adults.
• Smaller social networks were linked to poorer mental health in graded patterns.
• The smallest family and friend network size tripled the odds of distress.
• Similar graded patterns were observed for networks with professional contacts.
• Expanding social networks, including professional ties, is crucial for ageing.

## 1 Introduction

Japan is at the forefront of global population ageing. Nearly three in ten residents are now aged 65 years or older, and this proportion is expected to increase further in the coming decades (Statistics Bureau of Japan, 2024). Healthy ageing has therefore become a central policy concern, understood as maintaining physical, psychological and social well-being across the life course rather than merely avoiding disease (Rowe & Kahn, 1997; World Health Organization, 2021).

Social relationships are a core determinant of healthy ageing. A substantial body of research has shown that social integration and support are associated with lower mortality, reduced functional decline, better cognitive function and improved mental health (Berkman et al., 2000; Holt-Lunstad et al., 2010; Hawkley & Cacioppo, 2010; Steptoe et al., 2013). Conversely, social isolation and loneliness have been recognised as major public health challenges, particularly among older adults (National Academies of Sciences, Engineering, and Medicine, 2020; World Health Organization, 2021). In Japan, concerns about “kodokushi” (lonely deaths), shrinking multigenerational households and the weakening of neighbourhood associations have drawn attention to the social dimensions of late-life vulnerability.

Theoretical frameworks in social epidemiology and gerontology help explain why social connections matter for health. Berkman and colleagues (2000) conceptualise social networks as structural features that shape access to social support, social influence and engagement, which in turn affect health through behavioral, psychological and physiological pathways. The social convoy model (Antonucci & Akiyama, 1990) emphasises that individuals move through life surrounded by networks of family, friends and others who provide varying degrees of support. More recently, the “social cure” literature argues that group memberships and shared social identities can protect mental health by providing meaning, belonging and collective efficacy (Haslam et al., 2018, 2021). These perspectives converge in highlighting social relationships as powerful “non-medical” determinants of mental well-being.

Within this broad field, the specific role of social network size remains less clearly delineated. Many studies emphasise subjective or functional dimensions of social relationships—such as perceived support, loneliness, or relationship quality—which are clearly important for mental health (Kawachi & Berkman, 2001; Shankar et al., 2015). However, network size captures a different, structural dimension of social connectedness: the breadth of potentially available ties. This breadth matters because a larger network may provide more diverse opportunities for emotional, instrumental, and informational support, as well as more pathways for social participation and role continuity in later life (Lubben, 1988; Cohen et al., 1985; Cornwell & Waite, 2009; House et al., 1988). In this sense, network size does not directly measure whether relationships are emotionally satisfying or supportive in a subjective sense, but it does indicate the extent of a person's relational “convoy” and the range of people who may be available when support is needed. At the same time, small networks may reflect cumulative disadvantage, health-related withdrawal, or social exclusion.

Japan provides a particularly important context in which to examine these issues. Rapid demographic ageing has occurred alongside changes in family structures, including declining co-residence with adult children, and policy efforts to promote community-based integrated care. Digitalisation and persistent digital divides among older adults (Friemel, 2016; Czaja et al., 2019) may also shape how social networks are maintained or eroded. Studies from Japan have shown that social participation and social capital are associated with better mental health and lower mortality among older adults (Murayama et al., 2012; Steptoe et al., 2013; Takagi et al., 2013), yet relatively few have used nationwide data to examine graded associations between social network size and mental health using validated network scales such as the Lubben Social Network Scale (LSNS).

Understanding the social patterning of late-life distress has implications beyond descriptive epidemiology. If psychological distress is strongly associated with deficits in social connectedness, then part of what appears in clinical settings may reflect unmet social needs rather than problems best addressed only through medical treatment. This makes the question of social network size relevant to broader debates about overdiagnosis and medicalization. Classic and contemporary critiques have argued that an expanding range of human experiences has been redefined as medical disorders, often treated primarily through pharmacological or other clinical interventions (Illich, 1975; Conrad, 2007; Moynihan et al., 2012). Distress related to bereavement, role loss, neighbourhood context, or loneliness may therefore be framed primarily as mental illness rather than as a response to social conditions, potentially leaving the social determinants of distress insufficiently addressed. Against this backdrop, clarifying the extent to which social network deficits are associated with older adults’ mental health may help orient attention toward social and community-based strategies that complement, rather than simply extend, biomedical care.

In this study, we analysed data from the 2024 Japan COVID-19 and Society Internet Survey (JACSIS) to examine associations between social network size and mental health among adults aged 65–84 years. We used LSNS-6, which focuses on family and friends, and LSNS-8, which additionally includes professional contacts such as healthcare providers. Mental health was assessed using the CDC Healthy Days mental health item and the Kessler 6 (K6) scale for psychological distress. Our primary aim was to assess whether larger social network size was associated with better mental health and whether graded dose–response relationships were evident after adjustment for demographic, family-structure, socioeconomic, health, and lifestyle factors. We also compared LSNS-6 and LSNS-8 to examine whether including professional contacts altered the observed pattern of associations. Because the data are cross-sectional, the findings should be interpreted as associations only, and bidirectional relationships are plausible. A secondary aim, particularly relevant to debates about overdiagnosis and “too much medicine,” was to consider how these associations might inform efforts to address late-life distress through social rather than purely medical means.

## 2 Methods

### 2.1 Study design and data source

We conducted a cross-sectional analysis using data from the 2024 wave of the Japan COVID-19 and Society Internet Survey (JACSIS), an ongoing nationwide web-based survey designed to monitor social, economic, and health-related conditions in Japan (Tabuchi et al., 2020). Participants were drawn from a large online panel managed by a survey company, with quota sampling designed to approximate the national distribution of age, sex, and region. However, the panel is not a probability sample, and the resulting estimates should not be interpreted as nationally representative prevalence estimates.

For the present analysis, we restricted the sample to respondents aged 65–84 years. Among 28,000 respondents surveyed from December 2024 to January 2025, 6786 individuals met this age criterion and had complete data on the social network measures, mental health outcomes, and covariates described below. We used a complete-case approach. Because our primary focus was on associations rather than population prevalence, analyses were conducted using unweighted data while adjusting for a comprehensive set of demographic, family-structure, socioeconomic, health, and lifestyle variables. We acknowledge that this limits the generalizability of prevalence estimates and return to this point in the Limitations. The JACSIS 2024 protocol was approved by the relevant institutional ethics committees, and all participants provided electronic informed consent.

### 2.2 Measures

#### 2.2.1 Mental health outcomes

##### 2.2.1.1 Poor mental health days

Perceived mental health was assessed using the CDC Healthy Days mental health item: “During the past 30 days, for about how many days was your mental health not good?” (Centers for Disease Control and Prevention, 2000; Moriarty et al., 2003). Responses range from 0 to 30 days and represent the number of days with poor mental health.

To capture two substantively distinct contrasts, we derived two binary indicators from this item, consistent with prior use of the CDC Healthy Days framework in population health research (Centers for Disease Control and Prevention, 2000; Moriarty et al., 2003). The first contrasted respondents who reported no poor mental health days (0 days) with those who reported at least one poor mental health day (≥1 day), thereby identifying any deviation from optimal mental health during the past month. The second contrasted respondents who reported fewer than seven poor mental health days with those who reported seven or more days in the past 30 days, a threshold used in prior Healthy Days research to indicate more sustained poor mental health (Moriarty et al., 2003). For ease of interpretation in the regression models, these variables were coded so that a value of 1 indicated the better mental health category and 0 indicated the worse category.

##### 2.2.1.2 Psychological distress

Psychological distress was assessed using the Kessler 6 (K6) scale (Kessler et al., 2002), which comprises six items scored on a 5-point scale and summed to yield a total score from 0 to 24, with higher scores indicating greater distress. In this sample, internal consistency of the K6 was excellent (Cronbach's α = 0.90). We defined moderate psychological distress as K6 ≥5 versus <5, and severe psychological distress as K6 ≥13 versus <13. For these outcomes, we coded the presence of distress (moderate or severe) as 1 and the absence or low distress as 0, so odds ratios greater than 1 indicate higher odds of psychological distress (worse mental health).

#### 2.2.2 Social network size: LSNS-6 and LSNS-8

Social network size was measured using the Lubben Social Network Scale (LSNS) (Lubben, 1988; Lubben et al., 2006). The LSNS-6 comprises six questions—three concerning family or relatives and three concerning friends. These items ask how many such people the respondent sees or hears from at least monthly, feels at ease talking with about private matters, and feels close enough to call on for help. Each item is scored from 0 (“none”) to 5 (“nine or more”), yielding a total score from 0 to 30. Higher scores indicate larger and more engaged social networks. In our sample, internal consistency of the LSNS-6 was excellent (Cronbach's α = 0.97).

The LSNS-8 extends the LSNS-6 by adding two items on contact with professionals (e.g. healthcare providers, counsellors), producing a total score from 0 to 40. Internal consistency of the LSNS-8 was similarly high (α = 0.96). Although item responses refer to approximate numbers of individuals, the LSNS total should be interpreted as a summary scale score reflecting the breadth of available social ties rather than as a literal count of network members. This is because each item is recorded in ordered response categories and summed across multiple relational domains (Lubben, 1988; Lubben et al., 2006).

To examine graded associations across the observed distribution of social network size, we categorized both LSNS-6 and LSNS-8 total scores into tertiles. Tertile cut-points were determined empirically from the score distributions in the analytic sample by identifying the approximate 33rd and 67th percentile boundaries. For LSNS-6, this yielded categories of ≤7 points (low), 8–13 points (middle), and ≥14 points (high, reference category). For LSNS-8, the corresponding categories were ≤8 points (low), 9–14 points (middle), and ≥15 points (high, reference category). Unlike conventional LSNS cut-offs (e.g. <12 points for LSNS-6) developed for screening purposes, these tertiles were used to evaluate graded dose–response relationships within the analytic sample rather than to classify respondents according to a clinical threshold for social isolation. Accordingly, these categories should be interpreted as relative exposure groups within this sample. We acknowledge in the Limitations that they do not map directly onto screening thresholds used in clinical or public health practice.

#### 2.2.3 Covariates

Covariates were selected based on established social epidemiologic frameworks (House et al., 1988; Kawachi & Berkman, 2001; Umberson & Montez, 2010). Demographic, socioeconomic, and basic health covariates included sex (male or female; self-reported biological sex assigned at birth), age (years, continuous), marital status, educational attainment, household size, household annual income, household financial assets, BMI, current morbidity, and region.

Marital status distinguished respondents with a spouse from those without (including never married, divorced and widowed). Educational attainment was coded into four categories: high school or less; vocational school or junior college; university or graduate school; and other/do not know. Household size distinguished 1-person, 2-person, 3-person and ≥4-person households. Household annual income was categorized as <5 million Japanese Yen (JPY), 5–10 million JPY, ≥10 million JPY, and “prefer not to answer/do not know.” Household financial assets were categorized as <20 million JPY, ≥20 million JPY, and “prefer not to answer/do not know.” For both variables, “prefer not to answer/do not know” responses were retained as separate categories in the regression models in order to preserve sample size and make item non-response explicit. We acknowledge, however, that this approach does not eliminate the possibility of residual bias if non-response is socially patterned in ways not fully captured by the included covariates. BMI was calculated in kg/m^2^ (continuous). Current morbidity indicated the presence of any specified chronic physical or mental condition (e.g. diabetes, respiratory disease, cardiovascular disease, cancer, depression, other mental disorders). Region of residence was based on prefecture and grouped into eight macro-regions: Hokkaido, Tohoku, Kanto, Chubu, Kinki, Chugoku, Shikoku, and Kyushu/Okinawa. This grouping was used to capture broad regional differences while avoiding overparameterization.

Additional lifestyle and activity-related covariates comprised smoking history (current or past vs never), alcohol drinking history (any vs none), running or jogging habit (yes vs no), sports participation (yes vs no), and current working status (working vs not working).

Because marital status and household size were moderately correlated among older adults, we examined multicollinearity diagnostics and found no evidence of problematic variance inflation when both variables were included. Educational attainment is strongly related to lifetime socioeconomic position. In practice, including marital status and education in the models did not materially change the magnitude or direction of the associations between social network size and mental health; we therefore retained them in the main multivariable models and note that results are robust to their inclusion.

### 2.3 Statistical analysis

We first described sample characteristics by LSNS-6 tertiles, reporting means and standard deviations for continuous variables and counts and percentages for categorical variables. Differences across tertiles were tested using analysis of variance (ANOVA) for continuous variables and chi-squared tests for categorical variables.

For each mental health outcome and each measure of social network size (LSNS-6 and LSNS-8 tertiles), we fitted separate logistic regression models to estimate odds ratios (ORs) and 95% confidence intervals (CIs), with the highest tertile of network size as the reference group. We specified three models.

Model 1 included only the LSNS tertiles (unadjusted).
Model 2 additionally adjusted for demographic, socioeconomic, and basic health covariates (sex, age, marital status, educational attainment, household size, household annual income, household financial assets, BMI, current morbidity, and region).
Model 3 further adjusted for lifestyle and activity-related covariates (smoking history, alcohol drinking history, running/jogging, sports participation, and working status).

Because the Healthy Days outcomes were coded so that a value of 1 indicated better mental health, odds ratios below 1 represent lower odds of being in the better mental health category. By contrast, for the K6-based outcomes, a value of 1 indicated the presence of distress; therefore, odds ratios above 1 represent higher odds of worse mental health. We reiterate this direction in the relevant table titles and notes to facilitate interpretation.

To examine potential dose–response relationships, we tested for linear trends across LSNS tertiles by treating them as ordinal variables in Model 3. To address multiple comparisons arising from the examination of two LSNS measures across four mental health outcomes, we controlled the false discovery rate (FDR), that is, the expected proportion of false-positive findings among the rejected null hypotheses, using the Benjamini–Hochberg procedure (Benjamini & Hochberg, 1995). This adjustment was applied to the main Model 3 contrasts (middle vs high and low vs high) for each exposure–outcome combination. We report FDR-adjusted p-values in the main tables alongside the corresponding original p-values, and we summarize the raw p-values before FDR adjustment in Supplementary Table S1. Statistical significance was defined as a two-sided α = 0.05. Analyses were conducted using IBM SPSS Statistics, version 29.

## 3 Results

### 3.1 Sample characteristics

The analytic sample comprised 6786 older adults aged 65–84 years (mean 72.7 years, SD 4.9); 50.4% were men. Approximately 75% of respondents were married, and around 44% had completed university or graduate education. In terms of mental health, 11.8% of participants reported experiencing at least one poor mental health day in the past 30 days. Moderate psychological distress (K6 ≥5) was present in 16.3% and severe distress (K6 ≥13) in 1.9%. Table 1 presents detailed demographic, socioeconomic, health and lifestyle characteristics by LSNS-6 tertiles.

**Table 1:** Sample characteristics by Lubben Social Network Scale-6 (LSNS-6) tertiles (N = 6786).

Characteristic	High (n = 2134)	Middle (n = 2320)	Low (n = 2332)	*p*-value
**Demographic characteristics**				
Age, mean (SD), years	71.9 (4.7)	72.8 (4.9)	73.3 (5.0)	<0.001
Sex, male, n (%)	1225 (57.4)	1135 (48.9)	1063 (45.6)	<0.001
Marital status, married, n (%)	1699 (79.6)	1766 (76.1)	1641 (70.4)	<0.001
Educational attainment: high school or less, n (%)	745 (34.9)	828 (35.7)	891 (38.2)	0.001
Educational attainment: vocational school/junior college, n (%)	445 (20.9)	486 (20.9)	393 (16.9)	0.001
Educational attainment: university/graduate school, n (%)	937 (43.9)	1000 (43.1)	1033 (44.3)	0.001
Educational attainment: other/do not know, n (%)	7 (0.3)	6 (0.3)	15 (0.6)	0.001
Household size (1-person), n (%)	414 (19.4)	522 (22.5)	596 (25.6)	<0.001
Household size (2-person), n (%)	985 (46.2)	1059 (45.6)	1029 (44.1)	0.015
Household size (3 persons), n (%)	609 (28.5)	600 (25.9)	566 (24.3)	0.004
Household size (≥4 persons), n (%)	126 (5.9)	139 (6.0)	141 (6.0)	0.992
Household annual income (<5 million Japanese Yen (JPY)), n (%)	533 (25.0)	808 (34.8)	1088 (46.7)	<0.001
Household annual income (5–10 million JPY), n (%)	903 (42.3)	884 (38.1)	702 (30.1)	<0.001
Household annual income (≥10 million JPY), n (%)	698 (32.7)	628 (27.1)	542 (23.2)	<0.001
Household financial assets (≥20 million JPY), n (%)	659 (30.9)	470 (20.3)	308 (13.2)	<0.001
Body Mass Index (BMI), mean (SD), kg/m^2^	23.1 (3.5)	22.9 (3.6)	22.8 (3.7)	0.005
Current morbidity (yes), n (%)	1168 (54.7)	1328 (57.2)	1369 (58.7)	0.038
Region (Kanto), n (%)	779 (36.5)	832 (35.9)	785 (33.7)	0.052
**Lifestyle, exercise, and employment**				
Smoking history (yes), n (%)	1102 (51.6)	1159 (50.0)	1132 (48.5)	0.165
Drinking history (yes), n (%)	1304 (61.1)	1257 (54.2)	1104 (47.3)	<0.001
Running habit (yes), n (%)	379 (17.8)	355 (15.3)	305 (13.1)	<0.001
Sports habit (yes), n (%)	853 (40.0)	789 (34.0)	631 (27.1)	<0.001
Working status (working), n (%)	774 (36.3)	632 (27.2)	448 (19.2)	<0.001
**Mental health outcomes**				
Poor mental health (≥1 day), n (%)	198 (9.3)	253 (10.9)	352 (15.1)	<0.001
Moderate distress (Kessler Psychological Distress Scale-6 (K6) ≥ 5), n (%)	220 (10.3)	359 (15.5)	526 (22.6)	<0.001
Severe distress (Kessler Psychological Distress Scale-6 (K6) ≥ 13), n (%)	11 (0.5)	39 (1.7)	82 (3.5)	<0.001

Notes: Categorical variables are shown as n (%); continuous variables as mean (SD). p-values are based on chi-squared tests for categorical variables and ANOVA for continuous variables. LSNS-6 tertiles were defined as low (≤7), middle (8–13), and high (≥14; reference). For household annual income and household financial assets, respondents who selected “prefer not to answer/do not know” are not shown in the displayed rows (household income: n = 1,535, 22.6%; household financial assets: n = 2,616, 38.5%). Percentages for the displayed categories were calculated after excluding these responses from the denominators. In regression analyses, “prefer not to answer/do not know” was retained as a separate category for both variables. Current morbidity indicates the presence of any specified chronic physical or mental condition. Region is presented descriptively as Kanto versus other regions; in regression models, region was represented by eight macro-regions.

Participants in the highest LSNS-6 tertile were younger on average than those in the lowest tertile (mean age 71.9 vs 73.3 years) and were more often male (57.4% vs 45.6%; Table 1). They were also more likely to be married (79.6% vs 70.4%), less likely to live alone (19.4% vs 25.6%), and more likely to report higher household income and financial assets than those in the lowest tertile. Older adults in the highest tertile were also more likely to be working (36.3% vs 19.2%), to report alcohol drinking history (61.1% vs 47.3%), and to participate in running/jogging (17.8% vs 13.1%) and sports (40.0% vs 27.1%).

Mental health outcomes differed markedly across LSNS-6 tertiles. The proportion reporting at least one poor mental health day increased from 9.3% in the high LSNS group to 15.1% in the low group. Moderate psychological distress (K6 ≥5) rose from 10.3% in the high LSNS tertile to 22.6% in the low tertile, and severe distress (K6 ≥13) from 0.5% to 3.5% (p < 0.001 for all).

### 3.2 Social network size and poor mental health days

Table 2, Table 3 show the associations between LSNS tertiles and the two Healthy Days outcomes. Because these outcomes were coded so that higher values indicate better mental health, odds ratios below 1 indicate lower odds of being in the better mental health category.

**Table 2:** Association between Lubben Social Network Scale-6 (LSNS-6) and poor mental health days (N = 6786).

Outcome	Exposure (LSNS-6)	Model 1OR (95% CI)	*P*	Model 2OR (95% CI)	*p*	Model 3OR (95% CI) [FDR p]	*p* for trend
**Poor mental health days: 0 days vs ≥ 1 day (Healthy Days; higher is better)**							
0 days vs ≥ 1 day	Middle	0.891 (0.782–1.014)	0.081	0.903 (0.790–1.032)	0.134	0.919 (0.803–1.052) [0.219]	0.006
	Low	0.785 (0.690–0.892)	<0.001	0.762 (0.665–0.872)	<0.001	0.799 (0.695–0.918) [0.002]	
**Poor mental health days: < 7 days vs ≥ 7 days (Healthy Days; higher is better)**							
<7 days vs ≥ 7 days	Middle	0.787 (0.659–0.939)	0.008	0.785 (0.655–0.941)	0.009	0.812 (0.677–0.975) [0.026]	<0.001
	Low	0.641 (0.540–0.761)	<0.001	0.623 (0.521–0.746)	<0.001	0.689 (0.573–0.829) [0.000]	

Notes: LSNS-6 categories were high (≥14; reference), middle (8–13), and low (≤7). Model 1 was unadjusted. Model 2 was adjusted for sex, age, marital status, educational attainment, household size, household annual income, household financial assets, BMI, current morbidity, and region. Model 3 was additionally adjusted for smoking history, alcohol drinking history, running/jogging, sports participation, and working status. False discovery rate (FDR)-adjusted p-values are shown for Model 3 contrasts. For these Healthy Days outcomes, odds ratios below 1 indicate lower odds of being in the better mental health category.

**Table 3:** Association between Lubben Social Network Scale-8 (LSNS-8) and poor mental health days (N = 6786).

Outcome	Exposure (LSNS-8)	Model 1OR (95% CI)	P	Model 2OR (95% CI)	p	Model 3OR (95% CI) [FDR p]	p for trend
**Poor mental health days: 0 days vs ≥ 1 day (Healthy Days; higher is better)**							
0 days vs ≥ 1 day	Middle	0.802 (0.702–0.916),	0.001	0.840 (0.732–0.962)	0.012	0.859 (0.748–0.985) [0.030]	0.001
	Low	0.818 (0.722–0.928)	0.002	0.812 (0.712–0.926)	0.002	0.857 (0.749–0.981) [0.025]	
**Poor mental health days: < 7 days vs ≥ 7 days (Healthy Days; higher is better)**							
<7 days vs ≥ 7 days	Middle	0.724 (0.605–0.868)	<0.001	0.746 (0.620–0.896)	0.002	0.773 (0.642–0.931) [0.007]	<0.001
	Low	0.656 (0.554–0.777)	<0.001	0.641 (0.538–0.764)	<0.001	0.711 (0.594–0.851) [0.001]	

Notes: LSNS-8 categories were high (≥15; reference), middle (9–14), and low (≤8). Model 1 was unadjusted. Model 2 was adjusted for sex, age, marital status, educational attainment, household size, household annual income, household financial assets, BMI, current morbidity, and region. Model 3 was additionally adjusted for smoking history, alcohol drinking history, running/jogging, sports participation, and working status. False discovery rate (FDR)-adjusted p-values are shown for Model 3 contrasts. For these Healthy Days outcomes, odds ratios below 1 indicate lower odds of being in the better mental health category.

For the outcome of 0 versus ≥1 poor mental health day, smaller LSNS-6 network size was associated with lower odds of reporting 0 poor mental health days. In unadjusted models, the OR for the low LSNS-6 tertile compared with the high tertile was 0.79 (95% CI: 0.69–0.89). After adjustment for demographic, family-structure, socioeconomic and health factors (Model 2), and further for lifestyle and working status (Model 3), this association persisted (Model 3 OR = 0.80, 95% CI: 0.70–0.92, FDR p = 0.002). The OR for the middle tertile was closer to the null and not statistically significant after full adjustment (Model 3 OR = 0.92, 95% CI: 0.80–1.05, FDR p = 0.22), although the overall trend across tertiles remained significant (p for trend = 0.006).

For the outcome contrasting <7 versus ≥7 poor mental health days, graded associations were clearer. In LSNS-6 models, the low tertile had substantially lower odds of being in the better mental health category (<7 days) than the high tertile (Model 3 OR = 0.69, 95% CI: 0.57–0.83, FDR <0.001). The middle tertile also showed a significant disadvantage (Model 3 OR = 0.81, 95% CI: 0.68–0.98, FDR = 0.026), with a strong linear trend (p for trend <0.001) (raw p-values before FDR adjustment are shown in Supplementary Table S1).

LSNS-8 showed a similar pattern, although the estimates were somewhat closer to the null than those for LSNS-6. For 0 versus ≥1 poor mental health day, the fully adjusted ORs for the middle and low LSNS-8 tertiles were 0.86 (95% CI: 0.75–0.99, FDR = 0.030) and 0.86 (95% CI: 0.75–0.98, FDR = 0.025), respectively. For <7 versus ≥7 poor mental health days, the corresponding ORs were 0.77 (95% CI: 0.64–0.93, FDR = 0.007) and 0.71 (95% CI: 0.59–0.85, FDR <0.001), again with strong evidence of a dose–response trend.

### 3.3 Social network size and psychological distress

Table 4, Table 5 present the associations between LSNS tertiles and K6-based psychological distress. For these outcomes, a value of 1 indicates moderate or severe distress, so odds ratios greater than 1 reflect higher odds of distress (worse mental health) in the lower LSNS tertiles compared with the high tertile.

**Table 4:** Association between Lubben Social Network Scale-6 (LSNS-6) and psychological distress (N = 6786).

Outcome	Exposure (LSNS-6)	Model 1OR (95% CI)	*p*	Model 2OR (95% CI)	*p*	Model 3OR (95% CI) [FDR p]	*p* for trend
**Moderate psychological distress (K6 ≥5 vs < 5)**							
Moderate psychological distress	High (Ref.)	1.00	—	1.00	—	1.00	<0.001
	Middle	1.644 (1.371–1.972)	<0.001	1.589 (1.321–1.911)	<0.001	1.610 (1.337–1.938) [FDR<0.001]	
	Low	2.774 (2.336–3.294)	<0.001	2.686 (2.248–3.211)	<0.001	2.741 (2.284–3.288) [FDR <0.001]	
**Severe psychological distress (K6 ≥13 vs < 13)**							
Severe psychological distress	High (Ref.)	1.00	—	1.00	—	1.00	<0.001
	Middle	2.956 (1.498–5.836)	0.002	2.763 (1.395–5.471)	0.004	2.726 (1.375–5.405) [FDR 0.004]	
	Low	7.390 (3.934–13.881)	<0.001	6.935 (3.652–13.169)	<0.001	6.543 (3.423–12.506) [FDR <0.001]	

Notes: LSNS-6 categories were high (≥14; reference), middle (8–13), and low (≤7). Model 1 was unadjusted. Model 2 was adjusted for sex, age, marital status, educational attainment, household size, household annual income, household financial assets, BMI, current morbidity, and region. Model 3 was additionally adjusted for smoking history, alcohol drinking history, running/jogging, sports participation, and working status. False discovery rate (FDR)-adjusted p-values are shown for Model 3 contrasts. Odds ratios greater than 1 indicate higher odds of psychological distress.

**Table 5:** Association between Lubben Social Network Scale-8 (LSNS-8) and psychological distress (N = 6786).

Outcome	Exposure (LSNS-8)	Model 1OR (95% CI)	*p*	Model 2OR (95% CI)	*p*	Model 3OR (95% CI) [FDR p]	*p* for trend
**Moderate psychological distress (K6 ≥5 vs < 5)**							
Moderate psychological distress	High (Ref.)	1.00	—	1.00	—	1.00	<0.001
	Middle	1.391 (1.159-1.669)	<0.001	1.334 (1.109-1.606)	<0.001	1.352 (1.122-1.628) [0.002]	
	Low	2.309 (1.962-2.718)	<0.001	2.194 (1.854-2.596)	<0.001	2.231 (1.878-2.651)[<0.001]	
**Severe psychological distress (K6 ≥13 vs < 13)**							
Severe psychological distress	High (Ref.)	1.00	—	1.00	—	1.00	<0.001
	Middle	2.211 (1.161-4.213)	0.015	2.025 (1.059-3.873)	0.033	1.994 (1.041-3.819) [0.037]	
	Low	5.641 (3.203-9.936)	<0.001	5.165 (2.906-9.177)	<0.001	4.882 (2.726-8.741) [<0.001]	

Notes: LSNS-8 categories were high (≥15; reference), middle (9–14), and low (≤8). Model 1 was unadjusted. Model 2 was adjusted for sex, age, marital status, educational attainment, household size, household annual income, household financial assets, BMI, current morbidity, and region. Model 3 was additionally adjusted for smoking history, alcohol drinking history, running/jogging, sports participation, and working status. False discovery rate (FDR)-adjusted p-values are shown for Model 3 contrasts. Odds ratios greater than 1 indicate higher odds of psychological distress.

For moderate psychological distress (K6 ≥5), LSNS-6 showed a clear gradient. In unadjusted models, the ORs for the middle and low tertiles were 1.64 (95% CI: 1.37–1.97) and 2.77 (95% CI: 2.34–3.29), respectively. After full adjustment, associations remained strong (middle tertile: Model 3 OR = 1.61, 95% CI: 1.34–1.94, FDR <0.001; low tertile: OR = 2.74, 95% CI: 2.28–3.29, FDR <0.001). The p for trend was <0.001, indicating a robust dose–response pattern.

Associations were even more pronounced for severe psychological distress (K6 ≥13). Compared with the high LSNS-6 tertile, the fully adjusted OR for the middle tertile was 2.73 (95% CI: 1.38–5.41, FDR = 0.004), and the OR for the low tertile was 6.54 (95% CI: 3.42–12.51, FDR <0.001). Although the prevalence of severe distress was low, these odds ratios indicate substantial differences in risk between older adults with large versus very small networks.

Both LSNS measures showed clear graded associations with psychological distress, although the estimates were somewhat stronger for LSNS-6 than for LSNS-8. For moderate distress, the fully adjusted OR for the lowest versus highest tertile was 2.74 (95% CI: 2.28–3.29) for LSNS-6 and 2.23 (95% CI: 1.88–2.65) for LSNS-8. For severe distress, the corresponding ORs were 6.54 (95% CI: 3.42–12.51) for LSNS-6 and 4.88 (95% CI: 2.73–8.74) for LSNS-8.

### 3.4 Dose–response patterns

Across all outcomes and both LSNS measures, the fully adjusted models showed consistent dose–response relationships. As social network size decreased from the high to the middle to the low tertile, the odds of being in better mental health (based on Healthy Days) declined, and the odds of moderate and severe psychological distress increased. These graded patterns suggest that mental health benefits accrue across the spectrum of network size, not only at extremes of social isolation.

## 4 Discussion

This study examined the association between social network size and multiple mental health outcomes in a large, nationwide sample of older Japanese adults. We found that smaller social network size, as measured by both LSNS-6 and LSNS-8, was consistently associated with poorer mental health, including more poor mental health days and higher levels of psychological distress. These associations were graded across tertiles and remained robust after adjustment for demographic characteristics, marital status, education, socioeconomic conditions, morbidity, lifestyle factors and region.

### 4.1 Interpretation in light of existing literature and theory

Our findings are consistent with a broad literature showing that social integration and support are protective for mental health in later life (Kawachi & Berkman, 2001; Shankar et al., 2015; Steptoe et al., 2013). They also accord with prior Japanese research indicating that social participation and social capital are associated with better mental health in older populations (Murayama et al., 2012; Takagi et al., 2013), although direct comparison across studies is complicated by differences in age composition, survey mode, and outcome definitions. Our findings also align with the social convoy model (Antonucci & Akiyama, 1990), which frames social networks as dynamic convoys that accompany individuals through life's transitions. Within this framework, LSNS scores can be viewed as indicators of convoy size, and our results suggest that smaller convoys are associated with greater vulnerability to psychological distress, even when marital status, education, and material resources are taken into account.

The magnitude of some associations, particularly for severe distress, is striking. An adjusted OR of more than six for severe distress in the lowest LSNS-6 tertile implies a substantial difference in risk between older adults with very small versus large networks. Although severe distress is relatively rare, these differences suggest that network deficits may be an important contributor to late-life mental health inequalities, complementing other social determinants such as socioeconomic position and neighbourhood-level context (Murayama et al., 2012; Umberson & Montez, 2010).

The graded nature of the associations is also noteworthy. Rather than a simple threshold separating “isolated” from “non-isolated” individuals, mental health appears to improve progressively as network size increases. This resonates with the “social cure” perspective, which emphasises that each additional meaningful connection or group membership can incrementally strengthen social identity, belonging and perceived control (Haslam et al., 2018, 2021). From a policy standpoint, this suggests that interventions do not need to transform very small networks into very large ones to have impact; even modest expansions in network size may yield measurable mental health benefits.

Our comparison of LSNS-6 and LSNS-8 offers additional nuance. Including professional contacts in LSNS-8 did not fundamentally alter the associations, though effect sizes were somewhat smaller than for LSNS-6 in some models. This may reflect the predominance of informal ties with family and friends in older adults’ everyday support networks, with professional ties playing more specific roles during periods of illness or crisis. It may also indicate that the emotional and identity-related aspects of informal relationships are especially salient for outcomes such as daily mental health and K6-based distress.

### 4.2 The Japanese context: social structure and health policy

The Japanese context adds important nuance to these findings. Japan's rapid population ageing has unfolded alongside shifts in household structures, labour markets and community life. Declines in multigenerational co-residence, later retirement and urbanisation have reshaped older adults' opportunities to maintain and expand social networks. At the same time, policy emphasis on community-based integrated care in Japan has placed neighbourhoods, municipalities, and local organizations at the centre of strategies for supporting older residents.

Our results show that older adults with larger social networks tend to be better off socioeconomically, more physically active and more likely to be employed. This is consistent with evidence from Japan and other countries that social capital and social participation are socially patterned and linked to both physical and mental health (Murayama et al., 2012; Takagi et al., 2013; Umberson & Montez, 2010). It underscores that network deficits are not randomly distributed but reflect broader life-course inequalities. Older adults with fewer economic resources may face more barriers to participating in clubs, hobby groups or travel. Those with chronic illnesses or functional limitations may withdraw from social life, further shrinking their networks and potentially exacerbating distress in a bidirectional loop (Steptoe et al., 2013).

Digital divides further complicate this picture. Although we do not directly measure online connections, the use of a web-based survey implies that respondents had at least some digital access and literacy. Older adults who are digitally excluded are likely underrepresented in the sample, and may also be more socially isolated offline (Friemel, 2016; Czaja et al., 2019). Our estimates of both social network size and mental health may therefore be conservative for the broader older population. Yet even within this relatively connected group, the associations between network size and mental health are strong.

### 4.3 Implications for medicalization, hospital restructuring and “too much medicine”

The Special Issue's focus on overdiagnosis and medicalization invites reflection on how these findings intersect with current developments in Japanese health policy. Hospital bed reconfiguration – including reductions in acute-care beds and functional differentiation of hospital services – is underway as part of efforts to align bed supply with demographic and epidemiologic realities. At the same time, many national and public hospitals have reported persistent financial deficits, which has intensified debates about the “appropriate use” of hospital care and the sustainability of a system facing growing numbers of older patients.

Within this policy environment, mild to moderate late-life distress related to bereavement, loneliness, or shrinking social networks may sometimes come to clinical attention in ways that invite primarily medical responses, particularly if distress is framed mainly as an individual medical problem rather than as a response to social conditions. Classic critiques of medicalization and more recent work on overdiagnosis have warned that expanding diagnostic categories and the growing reach of medicine risk pathologizing everyday experiences and exposing people to unnecessary interventions (Illich, 1975; Conrad, 2007; Moynihan et al., 2012). Our findings do not directly measure diagnosis, treatment, or service use, but they do indicate that psychological distress is strongly associated with deficits in social networks even after controlling for morbidity and socioeconomic status. These implications should therefore be interpreted cautiously as broader public health inferences rather than direct evidence of medical overuse.

Seen from this perspective, part of what appears in clinical settings as “demand for more medicine” may in fact be the manifestation of unmet social needs. Responding to such distress solely through psychotropic medication or increased hospital contact risks adding to bed pressures and hospital deficits while leaving underlying social drivers intact. Strengthening social networks and community resources could therefore be an important complement to efforts to rationalise medical care and avoid “too much medicine.”

This perspective aligns with growing interest in social prescribing and other community-based interventions for older adults. By linking older people with small networks to local groups, volunteer opportunities, intergenerational programs, and peer-support initiatives, it may be possible to address some of the social roots of distress and to complement conventional clinical care with non-medical forms of support. In a context where hospital restructuring and fiscal sustainability are pressing concerns, investing in the social infrastructure of communities may be as important for mental health as investing in clinical infrastructure.

### 4.4 Public health and policy implications

From a public health standpoint, our findings suggest that interventions to support and expand older adults’ social networks should be integral to healthy ageing strategies in Japan and other super-ageing societies. Because the associations between network size and mental health are graded, even incremental increases in network size may be beneficial. Policies that lower financial and physical barriers to participation in community activities, support the development of age-friendly public spaces, and strengthen neighbourhood organizations are likely to have positive mental health effects.

In Japan, initiatives under the community-based integrated care system could explicitly incorporate social network enhancement as both a process and outcome indicator. Health and long-term care providers could work more closely with municipalities, non-profit organizations and citizen groups to identify older adults with small networks and to facilitate their participation in social activities. Social prescribing programs tailored to Japanese institutional and cultural contexts could enable clinicians to respond to late-life distress not only with prescriptions or referrals to specialist services, but also with links to social and community resources.

### 4.5 Strengths and limitations

This study has several strengths. It uses recent data from a large, nationwide survey in a super-ageing society, enabling examination of social networks and mental health in a context of particular policy relevance. It employs validated measures of social networks (LSNS-6 and LSNS-8) and psychological distress (K6), all of which showed excellent internal consistency in this sample. Our analytic models adjust for a wide range of demographic, family-structure, socioeconomic, health and lifestyle factors, including marital status and education, and we apply FDR control to address multiple comparisons. By examining both LSNS-6 and LSNS-8, and multiple mental health outcomes, we provide a nuanced picture of how network size relates to both daily mental health and psychological distress.

Important limitations should also be acknowledged. First, the cross-sectional design precludes causal inference. While we interpret smaller networks as a potential risk factor for poorer mental health, the reverse is also plausible: older adults experiencing distress may withdraw from social activities, leading to smaller networks. Longitudinal data are needed to clarify temporal ordering and to examine how changes in network size over time relate to onset, persistence and remission of distress (Hernán & Robins, 2020).

Second, the web-based, non-probability sample introduces potential selection bias. Older adults who are digitally excluded, of lower socioeconomic status, or in poorer health may be underrepresented. Because we used unweighted data, our prevalence estimates are not nationally representative. Moreover, the direction of bias may not be neutral: if digitally excluded older adults tend to have both smaller social networks and poorer mental health, the associations observed here may underestimate the strength of the underlying relationship in the broader older population. However, our primary focus was on associations, and we adjusted for key sociodemographic and health correlates of survey participation, which may mitigate some bias.

Third, LSNS scores primarily capture the quantity and frequency of social ties, not their quality. Large networks may include ambivalent or conflictual relationships, whereas smaller networks may be highly supportive. Loneliness, perceived social support and the subjective meaning of relationships were not assessed here but are likely to play important roles (Antonucci & Akiyama, 1990; Cacioppo et al., 2006).

Fourth, all data were self-reported, raising the possibility of recall and social desirability biases. Personality traits such as extraversion or neuroticism, which we did not measure, could influence both network reporting and mental health. In addition, our tertile-based categorization of LSNS-6 and LSNS-8 was chosen to assess graded associations across the sample distribution rather than to classify respondents using validated screening cut-offs for social isolation. These categories should therefore be interpreted as relative exposure groups within the analytic sample, not as clinical thresholds. We also retained “prefer not to answer/do not know” responses for household income and financial assets as separate categories in order to preserve sample size and make item non-response explicit in the models. Although this approach improves transparency, it does not eliminate the possibility of bias if socioeconomic non-response is patterned in ways not fully captured by the included covariates.

Finally, despite extensive adjustment, residual confounding by unmeasured social factors—including detailed marital histories, occupational trajectories, and neighbourhood-level characteristics—cannot be ruled out. Nevertheless, the robustness of associations across multiple specifications suggests that social network size is an important correlate of late-life mental health in its own right.

### 4.6 Future directions

Future research should use longitudinal designs to examine how changes in social network size over time relate to the onset, persistence, or remission of psychological distress, and to clarify potential bidirectional relationships. Studies should also move beyond network size alone to investigate how network quality, loneliness, perceived support, group-based identity processes, and cultural norms interact to shape mental health, and whether specific types of ties (family vs friends vs professionals) differentially contribute to well-being. It will also be important to assess whether these associations vary by sex, age, and digital access.

From the perspective of overdiagnosis and medicalization, qualitative and mixed-methods research could explore how older adults, families and healthcare professionals interpret and respond to psychological distress in the context of social isolation, and how these interpretations influence help-seeking, diagnosis and treatment. Such work would complement our quantitative findings and help refine social and clinical strategies for managing late-life distress in ways that avoid “too much medicine” while addressing the underlying social realities of ageing.

## 5 Conclusions

In a large national sample of older Japanese adults, smaller social network size was strongly and consistently associated with poorer mental health across multiple outcomes, with clear dose–response relationships. These associations remained robust after adjusting for marital status, education and other key covariates, and were observed for both LSNS-6 and LSNS-8. The findings reinforce the central role of social relationships in late-life mental well-being and suggest that benefits may accrue across the spectrum of network size, not only among those at the most extreme end of social isolation. This implies that healthy ageing strategies may need to move beyond threshold-based identification alone and also support incremental strengthening of everyday social ties.

In the context of hospital bed reconfiguration, financial strain on national hospitals and broader concerns about overdiagnosis and “too much medicine,” our results support approaches that prioritise social and community-based interventions as essential complements to biomedical care. In Japan's community-based integrated care context, practical next steps could include identifying older adults with socially small networks, strengthening referral pathways to local community resources, and improving coordination across health, long-term care, and community sectors. Such approaches may help address the social roots of psychological distress while complementing, rather than replacing, biomedical care.

## CRediT authorship contribution statement

**Kazumi Kubota:** Writing – original draft, Methodology, Formal analysis, Conceptualization. **Miya Aishima:** Writing – review & editing, Conceptualization. **Yukiko Matsumura:** Writing – review & editing, Conceptualization. **Takeshi Miura:** Writing – review & editing. **Maya Minamizaki:** Writing – review & editing. **Yu Koizumi:** Writing – review & editing. **Yuka Kanoya:** Writing – review & editing. **Takahiro Tabuchi:** Writing – review & editing, Supervision, Methodology, Data curation.

## Consent for publication

Not applicable.

## Availability of data and materials

The dataset analysed in this study is not publicly available due to governance arrangements for specially processed, de-identified information and participant privacy. Data may be available from the JACSIS steering group upon reasonable request and with necessary approvals (https://jacsis-study.jp/howtouse/). Initial inquiries should be directed to Dr Takahiro Tabuchi (tabuchitak@gmail.com).

## Ethics approval and consent to participate

The JACSIS 2024 survey was approved by the relevant institutional ethics committees [names and approval numbers omitted for double-anonymized review]. All participants provided electronic informed consent.

## Consent for publication

Not applicable.

## Availability of data and materials

The dataset analysed in this study is not publicly available due to governance arrangements for specially processed, de-identified information and participant privacy. Data may be available from the JACSIS steering group upon reasonable request and with necessary approvals (https://jacsis-study.jp/howtouse/). Initial inquiries should be directed to the corresponding author upon reasonable request.

## Ethics approval and consent to participate

The JACSIS 2024 survey was approved by the Shimonoseki City University Research Ethics Committee (acceptance no. 2509–0623) and the Tohoku University Graduate School of Medicine Ethics Committee (rapid review dated 26 March 2025; study number 2024-1-1035). All participants provided electronic informed consent.

## Declaration of generative AI and AI-assisted technologies in the manuscript preparation process

During the preparation of this work the author(s) used OpenAI's ChatGPT in order to edit and refine the English language of the manuscript. After using this tool/service, the author(s) reviewed and edited the content as needed and take(s) full responsibility for the content of the published article.

## Funding

This work was supported by the 10.13039/501100001691Japan Society for the Promotion of Science [grant numbers JP21H04856, JP25H01079, JP24K15576]; JST RISTEX [grant number JPMJRX21K6]; the National Institute for Environmental Studies; the Health Labor Sciences Research Grants [grant numbers 23EA1003, 22FA2001, 24LA0201, 22JA1005, 22FA1001, 22FA1010, 23EA1001, 23FA1004, 24FA1020, 24LA1002]; and the 10.13039/100009619Japan Agency for Medical Research and Development [grant number JP243fa627004].

The funders had no role in the design of the study; data collection, analysis or interpretation; manuscript writing; or the decision to submit the article for publication.

## Declaration of competing interest

The authors declare no competing interests directly related to this work. In the last 36 months, Dr Tabuchi has received research support, consulting fees or lecture fees from Daiichi Sankyo Healthcare Co., Ltd.; Johnson & Johnson K.K.; Data Seed Inc.; Workout-Plus LLC; and EMMA Co., Ltd. These entities were not involved in the study design; data collection, analysis or interpretation; manuscript writing; or the decision to submit the article for publication.

## Data Availability

Data will be made available on request.

## Abbreviations

CDC: Centers for Disease Control and Prevention
CI: Confidence interval
FDR: False discovery rate
JACSIS: Japan COVID-19 and Society Internet Survey
K6: Kessler Psychological Distress Scale-6
LSNS: Lubben Social Network Scale
OR: Odds ratio
