# Supplementary material for: Social network size and mental health among older adults in Japan: A nationwide cross-sectional analysis of the JACSIS 2024 study
Source: SSM Popul Health. 2026 Jun 24;35:101943. doi: 10.1016/j.ssmph.2026.101943 (PMC13321034; doi:10.1016/j.ssmph.2026.101943)
Supplement: Multimedia component 1 [file mmc1.docx]

**Supplementary Table S1. Raw p-values for main contrasts in Model 3 (before False Discovery Rate adjustment)**

| Lubben Social Network Scale-6 (LSNS-6) | | |  | Lubben Social Network Scale-8 (LSNS-8) | | |
| --- | --- | --- | --- | --- | --- | --- |
| Outcome / Contrast | Raw p-value | FDR-adjusted p-value |  | Outcome / Contrast | Raw p-value | FDR-adjusted p-value |
| Poor mental health days (0 vs ≥1 day) | | |  | Poor mental health days (0 vs ≥1 day) | | |
| Middle vs High | 0.219 | 0.219 |  | Middle vs High | 0.030 | 0.030 |
| Low vs High | 0.002 | 0.002 |  | Low vs High | 0.025 | 0.025 |
| Poor mental health days (<7 vs ≥7 days) | | |  | Poor mental health days (<7 vs ≥7 days) | | |
| Middle vs High | 0.026 | 0.026 |  | Middle vs High | 0.007 | 0.007 |
| Low vs High | <0.001 | <0.001 |  | Low vs High | <0.001 | <0.001 |
| Psychological distress, moderate (K6 ≥5) | | |  | Psychological distress, moderate (K6 ≥5) | | |
| Middle vs High | <0.001 | <0.001 |  | Middle vs High | 0.002 | 0.002 |
| Low vs High | <0.001 | <0.001 |  | Low vs High | <0.001 | <0.001 |
| Psychological distress, severe (K6 ≥13) | | |  | Psychological distress, severe (K6 ≥13) | | |
| Middle vs High | 0.004 | 0.004 |  | Middle vs High | 0.037 | 0.037 |
| Low vs High | <0.001 | <0.001 |  | Low vs High | <0.001 | <0.001 |

Notes: Raw two-sided p-values are from fully adjusted models (Model 3). Model 1: unadjusted. Model 2: adjusted for sex, age, marital status, educational attainment, household size, household annual income, household financial assets, BMI, current morbidity, and region. Model 3: additionally adjusted for smoking history, drinking history, running/jogging habit, sports participation, and working status. Contrasts are against the High tertile (reference): Middle vs High and Low vs High. False Discovery Rate (FDR)-adjusted p-values correspond to those reported in the main tables. Abbreviations: Lubben Social Network Scale-6 (LSNS-6); Lubben Social Network Scale-8 (LSNS-8); Kessler Psychological Distress Scale-6 (K6).
